# Pulmonary Embolism after Moderna Vaccination in Kidney Transplant Patients: Two Case Reports and Literature Review

**DOI:** 10.3390/vaccines10060868

**Published:** 2022-05-29

**Authors:** Wai-Nga Chan, Chih-Shou Chen, Dong-Ru Ho, Yun-Ching Huang, Jian-Hui Lin, Kuo-Tsai Huang, Yu-Liang Liu

**Affiliations:** Divisions of Urology, Department of Surgery, Chang Gung Medical Foundation, Chiayi City 613, Taiwan; ktwaiwaiwai@gmail.com (W.-N.C.); cv7589@cgmh.org.tw (D.-R.H.); peecorkspark@gmail.com (Y.-C.H.); jhl790424@gmail.com (J.-H.L.); uro056085@gmail.com (K.-T.H.); gsuro65818@gmail.com (Y.-L.L.)

**Keywords:** kidney transplantation, COVID-19, Moderna vaccine, pulmonary embolism, deep vein thrombosis, immunosuppressant

## Abstract

Currently, the coronavirus disease 2019 (COVID-19) pandemic is still an ongoing and constant medical issue, and with upcoming new variants, vaccinations and boosters remain important. The safety of vaccines in patients after kidney transplantation is an essential problem, with thrombosis being one of the severe side effects and vaccine-induced immune thrombotic thrombocytopenia (VITT) revealed as the most commonly reported syndrome for thromboembolic events following COVID-19 vaccination. Here, we present two cases of kidney transplantation developing pulmonary embolism post-Moderna vaccination within 30 days without thrombocytopenia. The first case was a 52-year-old man with history of type II diabetes, hypertension and hyperlipidemia who had had cadaveric kidney transplantation in September 2008, where right leg swelling with claudication occurred 23 days after the second Moderna vaccination. The second case was a 57-year-old man with history of type II diabetes and glaucoma who had had living-related kidney transplantation in April 2013 and then complained of exertional dyspnea 26 days after administration of the third Moderna vaccine. The advantages of vaccination even in immunocompromised patients far outweigh the disadvantages, although clinicians must understand the risks of deep-vein thrombosis or even pulmonary embolism for such patients, which might not occur after just the first vaccination.

## 1. Introduction

Currently, the COVID-19 pandemic is still an ongoing and constant medical issue, but with upcoming new variants, vaccinations and boosters remain important [1]. The safety of vaccines in patient after kidney transplantation, however, is an essential problem.

Common side effects after receiving the mRNA COVID-19 vaccine include pain or swelling at the injected site, tiredness, headache, fever, nausea or muscle pain. Thrombosis is one of the severe side effects after vaccination, with vaccine-induced immune thrombotic thrombocytopenia (VITT) being the most associated syndrome revealing the presence of platelet-activating antibodies against platelet factor 4 (PF4), a condition which clinically resembles severe heparin-induced thrombocytopenia [2,3,4,5,6]. Here we present two recipients who had previously undergone kidney transplantation and then developed pulmonary embolism without thrombocytopenia.

## 2. Detailed Case Description

### 2.1. Case 1

A 52-year-old man with history of type II diabetes mellitus, hypertension and hyperlipidemia had had cadaveric kidney transplantation in September 2008. His maintenance immunosuppressants were cyclosporin 25 mg/cap twice daily, mycophenolate sodium 180 mg/tab twice daily, methylprednisolone 4 mg/tab once daily and sirolimus 1 mg/tab once daily. He received 2 doses of the Moderna vaccine on the 19 November 2021 and the 23 December 2021.

Following this, he suffered from right low-extremity swelling and claudication 23 days after administration of the second vaccine, although no severe adverse effects were detected after the first dose. Medication was adjusted as mycophenolate sodium 180 mg from 1 piece twice daily to 2 pieces in the morning and 1 piece in the evening because of elevation of creatinine from 1.19 mg/dL to 1.35 mg/dL. Sirolimus level had been maintained at 6.6 ng/mL since September 2021, then mycophenolate sodium was changed back into 1 piece twice daily after December 2021 due to normal and stable creatinine. His average creatinine level in the past three months was 1.08 mg/dL, and the average sirolimus level was 7.1 ng/mL. Clinical characteristics during admission are shown in Table 1.

**Figure 1:**
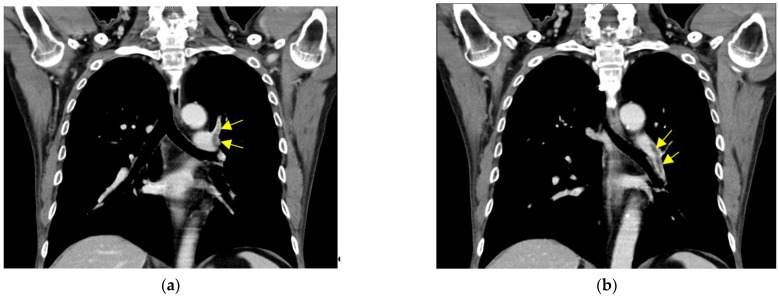

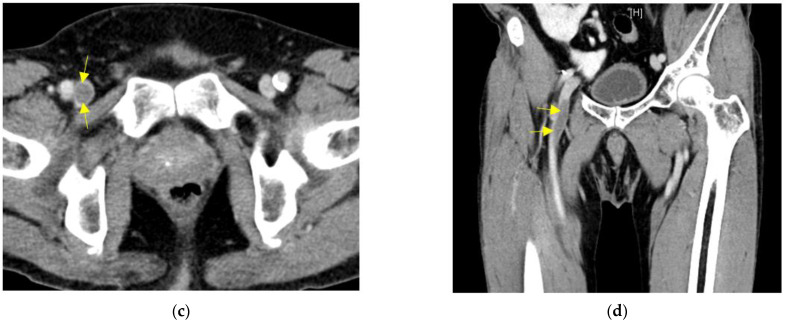
CT of chest and extremity: (**a**,**b**) left pulmonary artery thrombosis; (**c**,**d**) right femoral vein thrombosis. Arrow indicates thrombosis.

Computed Tomography (CT) of chest and extremities (Figure 1) showed left pulmonary artery thrombosis and right femoral vein thrombosis in the first case.

He was treated with enoxaparin twice daily following admission for 5 days, then this was changed to 60 mg endoxaban once daily. To date, his symptoms have improved without dyspnea.

### 2.2. Case 2

A 57-year-old man with history of type II diabetes mellitus and glaucoma had had living-donor kidney transplantation in April 2013. His maintenance immunosuppressants were everolimus 0.25 mg/tab twice per day, mycophenolate mofetil 250 mg/tab twice per day, methylprednisolone 4 mg/tab and cyclosporin 25 mg/cap twice per day, respectively. He had three doses of Moderna vaccines on 11 August and 22 November in 2021, then 15 January in 2022.

**Figure 2:**
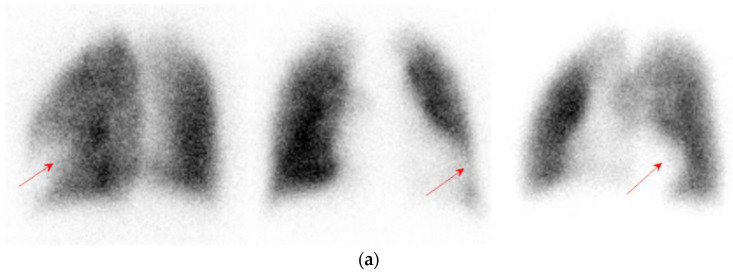

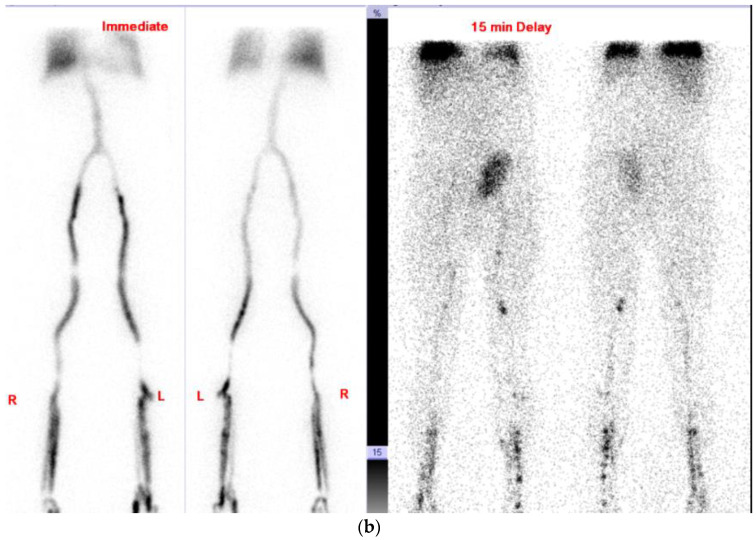
(**a**) Pulmonary perfusion scan showed a segmental cold area in the lingual inferior segment of left upper lobe, the arrow shows the defect; (**b**) Venography study showed well visualization of the deep veins in the bilateral lower extremities with good drainage of radioactivity up to the inferior vena cava.

Following the third dose, he complained of exertional dyspnea accompanied with bilateral leg edema 26 days later, although no severe adverse effects were detected after the previous two vaccinations. His average creatinine was 1.06 mg/dL, and average trough levels of cyclosporin and everolimus were 42.9 ng/mL and 4.3 ng/mL in the past three months, respectively. The clinical details when symptoms presented are shown in Table 1. He had not changed the immunosuppressants in the past 6 months; however, his drug level of cyclosporin had become elevated to 64.9 ng/mL with slight elevation of creatinine in the month he presented symptoms, so we tapered the cyclosporin to 25 mg daily. Pulmonary perfusion scan (Figure 2a) showed one segmental perfusion defect lesion at left lung revealed as a pulmonary embolism. Also, venography study (Figure 2b) showed negative for deep venous obstruction in bilateral low extremities.

He was treated at the outpatient department with a prescription for dabigatran 150 mg/cap twice daily and spironolactone 25 mg/tab daily. His symptoms have since improved during follow-up, and D-dimer has decreased to 0.19 mg/L.

In these two cases, they both had active social lives and lived independently. Their BMI were 22.9 and 22.3, respectively. The first case had quit smoking for the last 3 years, and the second case denied any smoking history; furthermore, they denied any recent trauma, personal or familial venous thromboembolism.

## 3. Discussion

We have 32 patients with kidney transplantation who have had various series of COVID-19 vaccinations (Table 2). Among these patients, of 30 who received at least one dose of Moderna vaccine, 16 patients (54%) had 3-dose vaccinations, 7 patients (23%) had 2-dose vaccinations, and the remaining 7 patients (23%) had 1-dose vaccinations. However, two of them (7%) developed thromboembolic events with pulmonary embolism and deep vein thrombosis without thrombocytopenia or severe side effects after previous Moderna vaccinations.

Thrombosis is one of the most severe adverse effects after vaccinations. Both Adenovirus-based vaccines (AstraZenaeca, Cambridge, UK; Johnson and Johnson, New Brunswick, NJ, USA) and mRNA-based vaccines (Pfizer, New York, NY, USA; Moderna) have been reported to cause thromboembolic events [5,7,8,9]. Vaccine-induced immune thrombotic thrombocytopenia (VITT) is the most reported associated syndrome of thromboembolic events with presentations of thrombocytopenia, thrombosis and disseminated intravascular coagulation. Tobaiqy, Mansour et al. [9] reported the most potential thrombotic adverse events resulted from the Oxford–AstraZeneca vaccine.

In fact, the Eudra Vigilance database showed that thromboembolic events due to vaccinations were 58.1% for Oxford–AstraZeneca, 32% for Pfizer and 9.8% for Moderna. Furthermore, thrombosis with thrombocytopenia has appeared in all three vaccines, with higher rates in those who had received the Oxford–AstraZeneca vaccine. Maria Abbattista et al. [8] demonstrated a lower rate of unusual site thrombosis for mRNA as compared to the adenoviral vaccines. Cerebral vein thrombosis along with thrombocytopenia has been reported for all vaccines, ranging from 3–5% to 31–44% for mRNA and adenoviral vaccines, respectively. Therefore, mRNA vaccines could be a safer choice for occurrence of thrombotic adverse events and appear less-associated to VITT as clinical presentation.

To our knowledge, calcineurin inhibitors (CNI-Cyclosporin and Tacrolimus), mycophenolate mofetil (MMF) and mammalian target of rapamycin inhibitor (mTORi-sirolimus and everolimus) are commonly used immunosuppressants. The CNI-induced thrombotic microangiopathy (TMA) produced a syndrome similar to thrombotic thrombocytopenic purpura (TTP) with potentially pathogenic inhibitory antibodies against the von Willebrand factor (vWF), causing cleavage of protease ADAMTS13 and leading to platelet aggregation [10,11]. A single-center cohort study demonstrated an increased incidence of TMA in the combination of cyclosporin (CsA) and silrolimus (SRL) at 20.7% compared to CsA + MMF (3.7%), Tacrolimus + SRL (6.1%) and Tacrolimus + MMF (1.3%) [12]. Only one case report reveals the linkage between MMF and thrombosis with factor V Leiden, so more definite studies are needed to establish the true relationship between DVT and MMF [13]. In consideration of VITT thromboembolic events, immunosuppressant-related thrombosis has also been recorded with a common presentation of TTP as reported more in cyclosporin-treated patients and in combination with CsA + SRL. However, drug induced TTP was less likely in our cases which did not have bleeding, anemia and thrombocytopenia, as they had not changed the dosages of CNI or mTORi in the past six months when the adverse events occurred.

Many studies have demonstrated a lower response rate among solid organ transplant patients than in the general population after vaccination [14,15]. In fact, COVID-19 infection after two doses of mRNA vaccines in kidney transplant patients has been reported in the real world [16].

As a consequence, clinicians should not neglect COVID-19 infection associated with thrombotic complications, especially in severe cases or inherited conditions [17]. Fiore JR et al. showed a case of rare homozygous prothrombin G20210A mutation in a young patient who developed severe pulmonary embolism and left iliac vein thrombosis after COVID infection [18]. In our two cases, the first case was tested before admission as per the policy of epidemic prevention in Taiwan government, but produced a negative result, although the second case was not tested because of outpatient department treatment protocol where no severe symptoms such as fever, headache, cough, rhinorrhea or diarrhea were presented.

In Taiwan, we have about 50 million vaccines that have been used already including 15 million doses of AstraZeneca vaccines (AZ), 17.5 million doses of Moderna vaccines, 15.5 million doses of BioNTech vaccines (BNT) and 2 million doses of MVC COVID-19 Vaccines (MCV) [19]. Overall reported severe adverse events from the Taiwan National Adverse Drug Reactions Reporting System number 8072 cases. Among all adverse events, 62 cases (62/50 million: 0.00012%) of idiopathic thrombocytopenic purpura (AZ for 44, Moderna for 14, BNT for 4) were reported with median age of 58.4 years and median onset of 14 days. Also, 71 cases (0.00014%) of thrombosis with thrombocytopenia syndrome (AZ for 59, Moderna for 6, BNT for 4 and MCV for 2) were reported with median age of 53.7 years and median onset of 13 days. Furthermore, 364 cases of all-thromboembolic events included 134 pulmonary embolisms (0.00027%) and 113 deep vein thrombosis incidences (0.00023%). On the other hand, 29 cases of cerebral venous sinus thrombosis (CVST) without thrombocytopenia were reported including AZ for 18, Moderna for 8, BNT for 2 and MCV for 1 with a median age of 51 years and median onset of 13 days [20]. CVST with thrombocytopenia has been more observed internationally; however, we also found CVST without thrombocytopenia clinically, and there might be a different mechanism in thrombosis after vaccination. Additionally, the incidence of non-thrombocytopenic thrombosis was 2.36% (2/86 injected doses), which was very high in our transplant series.

The mRNA vaccines were well tolerated and such severe adverse effects were not reported even in the third dose vaccination [21,22]. Our two cases demonstrated that thromboembolic events occurred even without adverse effects after the first Moderna vaccine. Clinicians should remain alert after every vaccination and recognize typical or atypical clinical manifestations of thromboembolic events for early diagnosis and adequate management in patients to reduce any critical damage. In these cases, Moderna-related thrombosis was not always associated with thrombocytopenia, meaning that there might be a different pathway for thrombosis in kidney transplant recipients receiving COVID-19 vaccinations and any judgement cannot be made merely on platelet counts.

## 4. Conclusions

Nowadays, the COVID-19 pandemic continues to spread with new variants. Protection through vaccinations is essential and another booster might be needed to increase the endogenous antibodies, although any risk of deep vein thrombosis or even pulmonary embolism for these immunocompromised patients must be understood, which might not occur after just the first vaccination.

On the other hand, for recipients of kidney transplantation already at risk of thrombosis by immunosuppressants, balanced evaluation of safety and potential severe adverse effects of vaccinations become even more critical issues with different considerations to the general population. Finally, VITT or TTP are not the only ways where thromboembolic events after COVID-19 vaccinations in kidney transplant patients who are under immunosuppressants therapies might develop. The higher incidence to have thromboembolic events in transplant patients might be the combined synergistic effects of immunosuppressants and COVID vaccinations.

## Figures and Tables

**Table 1:** Clinical characteristics of each patient.

Variable	Case 1	Case 2
Hemoglobin, g/dL	14.7	16.7
Platelet count, per μL	260,000	239,000
Creatinine, mg/dL	1.0	1.24
[Everolimus] *, ng/mL	/	6.4
[Sirolimus] *, ng/mL	6.8	/
[Cyclosporin] *, ng/mL	/	64.9
Troponin I, ng/mL	/	0.039
Pro-BNP, pg/mL	/	7231.51
D-dimer **, mg/L	3.59	1.10
Fibrinogen, mg/dL	439.5	396
Pulmonary embolism	Yes	Yes
other thrombosis	DVT ***	No
Symptom onset after vaccination, days	23 (after 2nd dose)	26 (after 3rd dose)
Anticoagulation therapy	Enoxaparin/Endoxaban	Dabigatran

[ ] * concentration of trough level. ** <0.5, 95% exclude pulmonary embolism. *** DVT: deep vein thrombosis.

**Table 2:** Types and vaccinations history in 32 recipients of kidney transplantation.

Vaccines	AstraZeneca	Moderna	BioNTech
**1st Dose**	6	24	2
**2nd Dose**	5	23	3
**3rd Dose**	/	22	1
**Accumulated Dose**	11	69	6

## Data Availability

Not applicable.
